# Biocompatibility and Antimicrobial Activity of *Reynoutria elliptica* Extract for Dental Application

**DOI:** 10.3390/plants9060670

**Published:** 2020-05-26

**Authors:** Song-Yi Yang, Min-Kyung Kang

**Affiliations:** 1 Department and Research Institute of Dental Biomaterials and Bioengineering, Yonsei University College of Dentistry, Seoul 03722, Korea; syyang88@yuhs.ac; 2 Department of Dental Hygiene, Hanseo University, Chungcheongnam-do 31962, Korea

**Keywords:** antibacterial, antifungal, cell viability, dental materials, *Reynoutria elliptica* extract, *Streptococcus mutans*, *Candida albicans*

## Abstract

This study was conducted to determine whether nature-derived *Reynoutria elliptica* extracts exhibit biocompatibility and antimicrobial effects against oral pathogens such as *Streptococcus mutans* and *Candida albicans*. Fine particles of *Reynoutria elliptica* extract were used to probe for biocompatibility and antimicrobial activity toward these pathogens*,* and results were evaluated with an MTT (3-[4,5-dimethylthiazol-2-yl]-2,5 diphenyl tetrazolium bromide) assay, spectrophotometric growth inhibitory assay, the total number of colony-forming units (CFU), an agar disk diffusion test, and scanning electron microscopy (SEM). In addition, UV/VIS spectroscopy was used to determine the levels of flavonoid and polyphenol in experimental solutions. Several experimental groups showed cell viability higher than 70%, and the antimicrobial activity toward both *S. mutans* and *C. albicans* was significantly higher than was that seen for the control group. In CFU and agar disk diffusion tests with *C. albicans*, increases in the concentration of *Reynoutria elliptica* extract led to significantly increased antimicrobial effects. Additionally, SEM results showed that *Reynoutria elliptica* extract changed the morphology and density of *S. mutans* and *C. albicans*. The results of this research can be applied to the use of *Reynoutria elliptica* extracts for the development of oral products that are biologically friendly and can control oral diseases such as dental caries and candida-associated denture stomatitis.

## 1. Introduction

Since the mouth is in direct contact with the external environment, it is constantly invaded by various microbes and has an environment suitable for bacterial growth and proliferation, both nutritionally and physiologically [1,2]. There are many kinds of microbes in human saliva and soft and hard tissue, and there are approximately 500 species that cause diseases. *Streptococcus mutans*, *Prevotella intermedia*, *Porphyromonas gingivalis,* and *Candida albicans* are among these, and the activities of these microbes in the mouth cause various oral diseases such as tooth decay, periodontal disease, oral candidiasis, and inflammation of the mouth [3,4,5,6]. In particular, oral diseases such as dental caries and periodontal diseases tend to leave permanent damage once they occur, so prevention, treatment, and post-treatment care are clearly important [7,8,9]. Therefore, inhibition of the activity of these oral pathogens is significant in the prevention and treatment of oral diseases [10].

*Streptococcus mutans* contributes to the rigid attachment of insoluble glucans to the tooth surface through glucosyltransferase action [11,12]. *Streptococcus mutans* is also known to adhere to the tooth surface and cause biofilm formation, which breaks down sugars and starches to release acids that may lead to tooth caries [13]. Thus, to prevent tooth caries, the use of antimicrobial materials is required to suppress the growth of *S. mutans* or to inhibit the attachment of other bacteria [14,15,16]. In addition, *C. albicans* is a common cause of oral mucosal infections, in which resident microbes in the mouth cause disease in patients with weakened immune systems and attach to the oral epithelial tissue or dentures. This often results in opportunistic infection and oral candidiasis [17,18,19]. Antifungal treatments with topical agents or medications are often utilized to treat these oral diseases and improve oral hygiene [20,21,22,23].

Prevention of oral diseases requires control of the formation of biofilms in the oral environment. Prior studies have been conducted to prevent and treat oral diseases using chlorhexidine, sodium lauryl sulfate, triclosan; moreover, antibiotics such as ampicillin, erythromycin, and penicillin are widely used in the dental clinic to suppress microbial growth [24,25,26,27,28]. However, it is known that the continuous use of synthetic chemicals and antibiotics with conventional antimicrobial properties can cause side effects such as cytotoxicity and mutagenicity. These result from the accumulation of synthetic antibacterial substances and, in the case of excessive use of these substances, changes in oral microbial strains, and both normal microbial residents and pathogens can be eliminated in the oral cavity [29,30,31]. Therefore, other publications have reported that limited use of antifungal agents is required to maintain host health while controlling harmful microbial pathogens [32,33].

Recently, many natural medicines, which have fewer side effects and offer the possibility of long-term use, have been evaluated to determine their antibacterial and anti-inflammatory potential [34,35,36,37,38]. Included among these is a large group of perennial plants in the Polygonaceae, which are used in East Asian medicine to promote blood circulation, relieve pain, treat diuretic and menstrual disorders, and alleviate respiratory problems. In addition, other prior studies have demonstrated that *Reynoutria elliptica* has excellent antimicrobial activity against *Helicobacter pylori*, and other studies indicated that it contained a growth inhibitor for *S. mutans* and thus explored its ability to prevent dental caries [39,40,41,42,43].

However, there have been no scientific studies on the biocompatibility and antimicrobial properties of various concentrations of these naturally derived *Reynoutria elliptica* extracts. Therefore, we discuss herein the potential use of the natural substance *Reynoutria elliptica* extract for application in antibacterial and antifungal oral products. The purpose of this study is to evaluate the biocompatibility using L929 fibroblast cells and determine the antimicrobial activity against *S. mutans* and *C. albicans* strains, and also to provide basic data on *Reynoutria elliptica* extracts with the use of component analysis. The null hypothesis of this study is that various concentrations of the *Reynoutria elliptica* extract will not exhibit significant biocompatibility or antimicrobial properties.

## 2. Materials and Methods

### 2.1. Preparation of Reynoutria elliptica Extracts

*Reynoutria elliptica* grown in North Gyeongsang province located in Korea was purchased from an herbal shop (Hanyakjae market, Seoul, Korea). Five hundred grams of *Reynoutria elliptica* was crushed, poured into 5 L of a 70% methanol solution, and extracted at room temperature (25 ± 1 °C) for 2 days. This extract solution was filtered through #2 filter paper (Whatman, Maidstone, UK) and was then evaporated and concentrated with a vacuum evaporator (EYELA, Tokyo, Japan). To produce the dried solid form, concentrated *Reynoutria elliptica* extract was frozen at −20 °C for 12 h and then stored in a freeze dryer (Ilshin Lab Co., LTD, Gyeonggi-do, Korea) at −55 °C for 48 h. The freeze dried *Reynoutria elliptica* extract was ground to a fine powder using a mortar and pestle. The resulting *Reynoutria elliptica* extract powder was stored in a desiccator at room temperature (25 ± 1 °C) before subsequent testing.

### 2.2. Preparation of Cells for Biocompatibility Tests

L929 mouse fibroblast cells were chosen to determine the biocompatibility of the *Reynoutria elliptica* extract. The cells were cultured as a monolayer in 75 T-flasks (SPL Life Science, Gyeonggi-do, Korea), sub-cultured three times within a week at 37 ± 1 °C in a humidified 5% CO_2_/air environment and maintained at the fifth passage. The cell culture medium used was RPMI 1640 (Gibco Laboratories, Grand Island, NY, USA) with 10% (*v/v*) fetal bovine serum (Gibco Laboratories, Grand Island, NY, USA) and 1% antibiotic-antimycotic solution (Anti-Anti, Gibco Laboratories, Grand Island, NY, USA). Cells adhering to the flask were detached with a mixture of 0.05% Trypsin-EDTA (Trypsin-EDTA, Gibco Laboratories, Grand Island, NY, USA), incubated for 10 min at 37 ± 1 °C, and used for cell inoculation. One hundred microliter of a cell suspension with 1 × 10^5^ cells/mL were then seeded in 96-well culture plate (Thermo Fisher Scientific Inc., Waltham, MA, USA) and incubated at 37 ± 1 °C in a humidified 5% CO_2_/air environment for 24 h to enable attachment.

### 2.3. Cell Viability Assay

The *Reynoutria elliptica* extract powder was irradiated for 30 min under ultraviolet light to prevent contamination, and then sterilized *Reynoutria elliptica* extract powders were dispersed with varying concentrations. To prepare the experimental solution, *Reynoutria elliptica* extract powders were dissolved in RPMI 1640 cell culture medium to achieve concentrations of 50 μg/mL, 100 μg/mL, 150 μg/mL and 200 μg/mL, and these solutions were stored at 37 ± 1 °C in a humidified 5% CO_2_/air environment for 24 h (in accordance with ISO standard 10993-12). In addition, RPMI 1640 cell culture medium that did not contain *Reynoutria elliptica* extract was also stored as described above to establish a negative control for cell viability tests.

A test for in vitro cytotoxicity, the MTT assay, was performed according to ISO 10993-5. The culture medium in the 96-well plate was removed from the wells, and 100 μL of the experimental and control solution was added into the each well. The plates were further incubated for 24 h, and the test and control solutions were removed. Then, each well was washed with 100 μL of pre-warmed Dulbecco’s phosphate-buffered saline solution (D-PBS, Gibco Laboratories, Grand Island, NY, USA). The D-PBS was removed, and the wells were refilled with 50 μL of 1 mg/mL MTT-tetrazolium salts (Sigma-Aldrich, St. Louis, MO, USA) in PBS and the plates were kept at 37 ± 1 °C in a humidified 5% CO_2_/air environment for 2 h in the dark. The MTT solution was then removed, and 100 μL of isopropanol (Sigma-Aldrich, St. Louis, MO, USA) was added to each well. The plates were then shaken on a shaker for 20 min in the dark. Subsequently, the optical density was measured using an ELISA reader (Epoch, BioTek, Winooski, VT, USA) at 570 nm. Cell viability was calculated using the following equation: Cell viability (%) = (Optical density of treated cell / Optical density of negative control) × 100. Cell viability in the negative control group was considered to be 100%, and the percentage values for the experimental groups were calculated. The MTT assay was repeated in five separate experiments.

Additionally, the morphology of the L929 cells on the 96-well plate after exposure to experimental and control solutions for 24 h was observed visually using an EVOS FL microscope (EVOS FL, Advanced Microscopy Group USA Ltd., Mill Creek, WA, USA) at 20 × magnification (n = 2).

### 2.4. Preparation of Microbial for Antimicrobial Test

To determine the antimicrobial potential of various concentrations of *Reynoutria elliptica* extract, *Streptococcus mutans* (ATCC 25175) and *Candida albicans* (ATCC 10231) were selected as test microorganisms. *Streptococcus mutans* was cultured in brain heart infusion (BHI, Becton Dickinson and Co., MD, USA) and *C. albicans* in yeast mold (YM, Becton Dickinson and Co., Franklin Lakes, NJ, USA), and then both were incubated at 37 °C for 24 h under aerobic conditions.

### 2.5. Antimicrobial Tests

To prepare the experimental solution, *Reynoutria elliptica* extract powder was dissolved in dimethyl sulfoxide (DMSO, Sigma-Aldrich, St. Louis, MO, USA) to achieve concentrations of 50 μg/mL, 100 μg/mL, 150 μg/mL and 200 μg/mL. In addition, DMSO without *Reynoutria elliptica* extract was also prepared to establish a negative control for the antimicrobial tests.

To study growth inhibition in a culture medium, microbial suspensions were diluted with each culture solution to establish an OD value within the range of 0.4–0.6 at 600 nm. Subsequently, the experimental solution and microbial suspension was mixed in a 9:1 ratio and incubated at 37 °C for 6 h, 12 h, and 24 h. A reaction mixture containing no *Reynoutria elliptica* extract was also cultured under the same conditions to prepare the control group. Then, the growth inhibitory effect of the experimental groups was estimated by using an ELISA reader to measure OD values (600 nm) after the three different time intervals.

To examine the microbial colony-forming unit (CFU), each experimental solution was added into microbial suspensions (1 × 10^5^ cells/mL) at a 1:1 ratio and incubated at 37 °C for 24 h. A reaction mixture without *Reynoutria elliptica* extract was also cultured under the same conditions to establish the control group. One hundred microliter of this mixture was then spread onto BHI and YM agar plates and incubated at 37 °C for 24 h, respectively. After incubation, the total viable cells were counted according to the number of CFUs.

To perform the agar disk diffusion test, 100 μL of microbial suspension (1 × 10^4^ cells/mL) was spread uniformly onto BHI and YM agar plates. Twenty microliters of the experimental solution were impregnated into a sterile paper disk with a diameter of 10 mm and thickness of 1 mm, and this was then placed on the surface of the agar plates, which had been inoculated with *S. mutans* and *C. albicans* suspension, respectively. Blank disks impregnated with DMSO were used as a negative control and also placed with the experimental group. The plates were then incubated for 24 h at 37 °C, and the size of the inhibition zone around each sample was measured with Vernier calipers (Mitutoyo, Kawasaki, Japan), with an accuracy of ± 0.01 mm.

To examine further the effect of the *Reynoutria elliptica* extract on microbial morphology, 0.5 mL of each experimental solution was added into microbial suspensions (1 × 10^5^ cells/mL) at 1:1 ratios. Subsequently, the 1 mL of the microbial suspension and experimental solution was added to a 24-well plate (Thermo Fisher Scientific Inc., Waltham, MA, USA) and incubated at 37 °C for 24 h. For microscopic examination, the *S. mutans* and *C. albicans* were fixed with 2% glutaraldehyde/paraformaldehyde in 0.1 M PBS for 30 min at room temperature (25 ± 1 °C). The fixed *S. mutans* and *C. albicans* were then post-fixed with 1% OsO_4_ dissolved in 0.1 M PBS for 2 h, dehydrated with an ascending gradual series of ethanol, treated with isoamyl acetate, and then subjected to critical point drying (LEICA EM CPD300; Leica, Wien, Austria). The *S. mutans* and *C. albicans* was then sputter-coated with Pt and observed using a scanning electron microscopy (FE-SEM; Merin, Carl Zeiss, Oberkochen, Germany) with an accelerating voltage of 2.0 kV.

All antimicrobial experiments except for the SEM observations were independently performed with five repetitive tests for each microbial strain, and data were recorded as means and standard deviations.

### 2.6. Extract Analysis

To analyze the extracts from the experimental solution, *Reynoutria elliptica* extract powder was dissolved in distilled water to achieve the concentrations 50 μg/mL, 100 μg/mL, 150 μg/mL, and 200 μg/mL, and the solutions were then held at 37 ± 1 °C in a water bath for 24 h.

To quantify the polyphenol content, 50 μL of the extract experimental solution and 50 μL of Folin Denis reagent were added to 650 μL of distilled water and allowed to react at room temperature (25 ± 1 °C) for 3 min. One hundred microliter of 10 % Na_2_CO_3_ solution and 150 μL of distilled water were added to the reacted solution to establish a final volume of 1 mL. These pretreated extracts were stored at 37 ± 1 °C for 60 min in a dark environment. The absorbance at 725 nm was subsequently determined using a UV/VIS spectrophotometer (X-ma 1200 Spectrophotometer, Human Corp., Seoul, Korea). Gallic acid (Sigma–Aldrich, St Louis, MO, USA) was used as a standard material to construct a calibration curve (20, 40, 60, 80, and 100 μg/mL). Using the calibration curve created with standard material, the total polyphenol content was calculated in μg of gallic acid equivalents.

To quantify the flavonoid content, 100 μL of the experimental solution and 1 mL of diethylene glycol was added to 100 μL of 1N sodium hydroxide and allowed to react at a temperature of 37 ± 1 °C for 60 min. The absorbance at 420 nm was subsequently determined with a UV/VIS spectrophotometer. Naringin (Sigma–Aldrich, St Louis, MO, USA) was used as a standard material to construct the calibration curve (20, 40, 60, 80, and 100 μg/mL). Using the calibration curve created with standard material, the total flavonoid content was calculated in μg of naringin equivalents.

### 2.7. Statistical Analysis

All test results from the control and experimental groups were analyzed with one-way ANOVA (PASW 18.0, IBM Co., Armonk, NY, USA) to confirm the interactions with various concentrations of *Reynoutria elliptica* extract. To determine significant differences within these concentrations, post-hoc analyses were carried out with Tukey’s multiple comparison test at a significance level of 0.05.

## 3. Results

### 3.1. Cell Viability and Morphology

**Figure 1:**
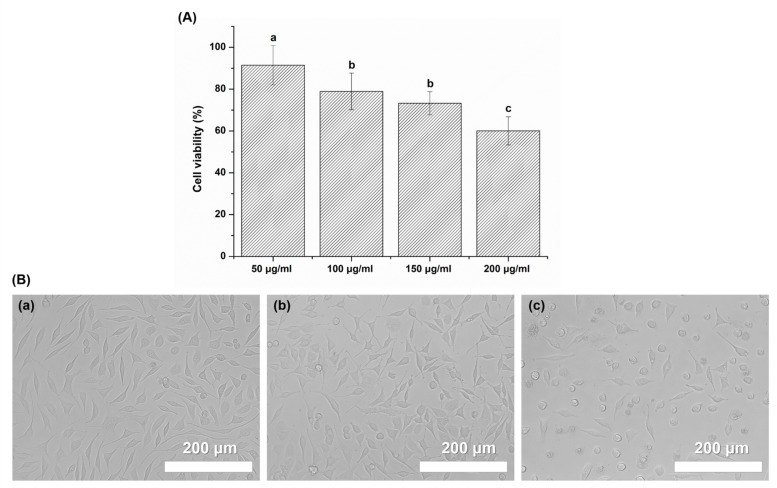
(**A**) Cell viability of each experimental groups (50 μg/mL, 100 μg/mL, 150 μg/mL and 200 μg/mL). The same lowercase letter indicates no significant differences in cell viability between the group (*p* > 0.05). (**B** (**a**)) L929 cells exposed to negative control for 24 h. (**B** (**b**)) L929 cells exposed to *Reynoutria elliptica* extract at a concentration of 50 µg/mL for 24 h. (**B** (**c**)) L929 cells exposed to *Reynoutria elliptica* extract at a concentration of 200 µg/mL for 24 h.

One-way ANOVA revealed significant differences in cell viability among the different concentration groups (*p* < 0.05) (Figure 1A). The results exhibited decreasing levels of cell viability with increasing concentrations of *Reynoutria elliptica* extract, and the 200 μg/mL group presented the lowest cell viability value (*p* < 0.05). The 50 μg/mL group had a cell viability of 91.5 ± 9.4 %, which was significantly higher than that of the other groups (*p* < 0.05). The groups with 100 μg/mL and 150 μg/mL concentrations exhibited no significant difference (*p* > 0.05) in cell viability. Additionally, all experimental groups (except for the 200 μg/mL group) showed cell viability greater than 70%, and these values are considered to indicate non-cytotoxic behavior (based on ISO biocompatibility criteria).

With a decreasing concentration of *Reynoutria elliptica* extract, the cells demonstrated the typical stellate appearance of the L929 cells (Figure 1B). The cells in the 50 µg/mL group showed a morphological appearance similar to that of the control group, but revealed a reduced tendency, relative to the control group, to attach to the surface of the culture plate. When the concentration of the *Reynoutria elliptica* extract was increased, the cells showed a toxic response; the cells in the 200 μg/mL group became rounded and lost the structural organization seen in the control group.

### 3.2. Optical Density

**Figure 2:**
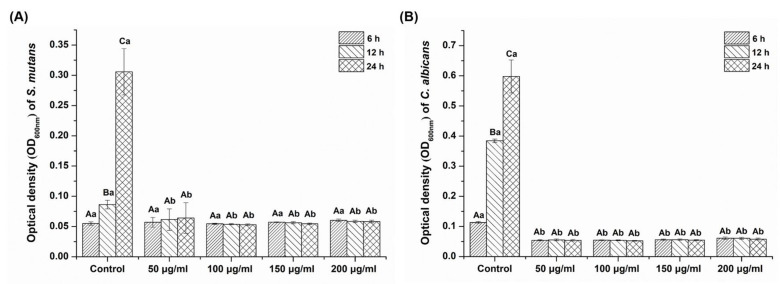
Optical density (OD_600nm_) of each experimental group (50 μg/mL, 100 μg/mL, 150 μg/mL and 200 μg/mL) with (**A**) *Streptococcus mutans* and (**B**) *Candida albicans* at three different times, compared with a control group. The same lowercase letter (**a**–**b**) indicates no significant differences in optical density between the group at each time point (*p* > 0.05). The same uppercase letter (**A**–**C**) indicates no significant differences in optical density value among three different times for each group (*p* > 0.05).

Figure 2 shows that the *Reynoutria elliptica* extract significantly affected the OD values for both *S. mutans* and *C. albicans,* as compared with the control group (*p* < 0.05). The control group showed higher OD values at various cultivation times than did the experimental groups (*p* < 0.05), with the only exception being the S. *mutans* sample cultivated for 6 h. The OD values for the *S. mutans* and *C. albicans* experimental groups at various cultivation times were not significantly different, despite the increase in the *Reynoutria elliptica* extract concentration (*p* > 0.05).

As shown in Figure 2, there were no differences in OD values for any experimental group containing *Reynoutria elliptica* extract. Conversely, the OD values for the control group without *Reynoutria elliptica* increased nearly 6-fold between the 6- and 24-h times for both *S. mutans* and *C. albicans* (*p* < 0.05). These results demonstrate that the *Reynoutria elliptica* extract significantly inhibited the growth of *S. mutans* and *C. albicans.*

### 3.3. CFU

**Figure 3:**
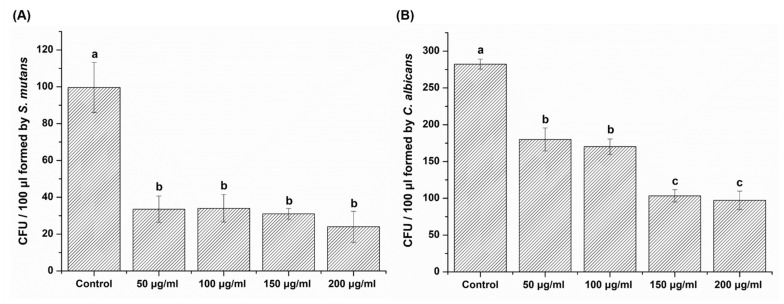
Colony-forming units (CFU) number for each experimental group (50 μg/mL, 100 μg/mL, 150 μg/mL and 200 μg/mL) with (**A**) *Streptococcus mutans* and (**B**) *Candida albicans,* compared with a control group. The same lowercase letter (**a**–**c**) indicates no significant difference in CFU values between the groups of a given microbial (*p* > 0.05).

One-way ANOVA revealed that the CFU of the experimental groups was significantly lower than was that of the control group with both *S. mutans* and *C. albicans* (*p* < 0.05) (Figure 3). However, the CFUs for *S. mutans* did not differ significantly among experimental groups, despite the increase in the *Reynoutria elliptica* extract concentration (*p* > 0.05). In the case of *C. albicans*, the results showed a decreasing trend for CFU with an increasing concentration of *Reynoutria elliptica* extract. The CFU value for the 150 μg/mL sample (103.25 ± 8.42) was not significantly different from that of the 200 μg/mL sample (97.25 ± 12.45) (*p* > 0.05). Additionally, the CFU for the 50 μg/mL sample (180.00 ± 15.60) was not significantly different from that of the 100 μg/mL sample (170.25 ± 10.44) (*p* > 0.05). These results demonstrated that *Reynoutria elliptica* extract significantly inhibited colony formation with *S. mutans* and *C. albicans.*

### 3.4. Growth Inhibition on Agar Plates

**Figure 4:**
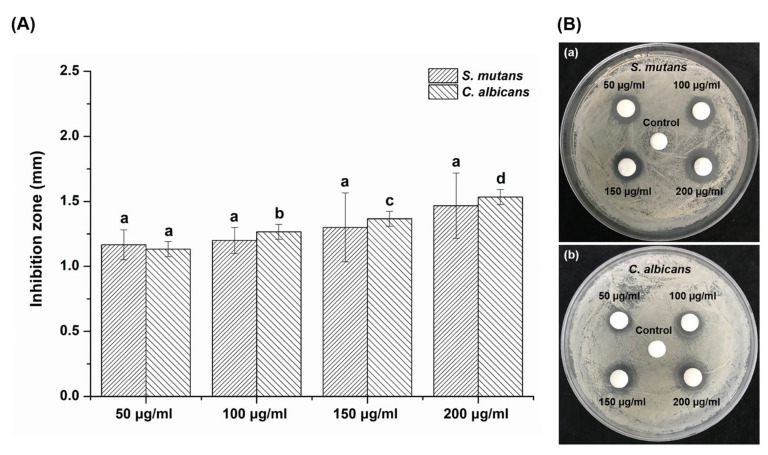
(**A**) Inhibition zone size for each experimental group (50 μg/mL, 100 μg/mL, 150 μg/mL and 200 μg/mL) on *Streptococcus mutans* and *Candida albicans* agar plates. The same lowercase letter (a–d) indicates that there was no significant difference in inhibition zone sizes for the experimental groups of the two microbial (*p* > 0.05). (**B**) Images of the inhibition zones for various concentrations of *Reynoutria elliptica* extract with (**a**) *Streptococcus mutans* and (**b**) *Candida albicans* agar plates.

The antimicrobial activities for different concentrations of *Reynoutria elliptica* extract were screened with the agar disk diffusion method, which is a technique for confirming antimicrobial potency according to the size of a transparent circle formed by restraining the growth of microbials around experimental materials on agar plates. The inhibition zones for different experimental groups did not differ significantly, despite increases in the *Reynoutria elliptica* extract concentrations on the *S. mutans* agar plate (*p* > 0.05) (Figure 4A). In contrast, the inhibition zone was significantly enlarged with higher concentrations of the *Reynoutria elliptica* extract on the *C. albicans* agar plate (*p* < 0.05).

Figure 4B shows microbial static rings produced around the experimental disks, which indicated that *Reynoutria elliptica* extract had an antimicrobial effect on both *S. mutans* and *C. albicans*. DMSO-treated disks used as negative controls (spot in the middle of the plate) showed no zone of inhibition with either *S. mutans* or *C. albicans*.

### 3.5. Morphological Observations Using SEM

**Figure 5:**
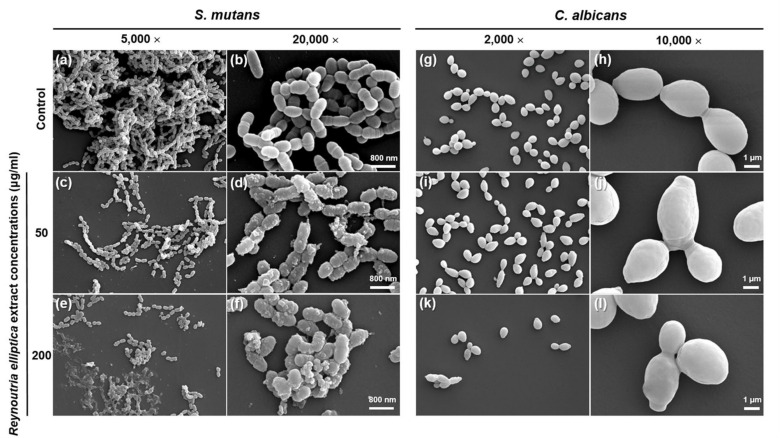
Scanning electron micrographs of *Streptococcus mutans* and *Candida albicans* treated with various concentrations of *Reynoutria elliptica* extract for 24 h. For *Streptococcus mutans* with *Reynoutria elliptica* extract, images (**a**) and (**b**) show to the control group that was not exposed to *Reynoutria elliptica* extract. Images (**c**) and (**d**) show *Streptococcus mutans* exposed to 50 μg/mL of *Reynoutria elliptica* extract. Images (**e**) and (**f**) show *Streptococcus mutans* exposed to 200 μg/mL of *Reynoutria elliptica* extract. For *Candida albicans* with *Reynoutria elliptica* extract, images (**g**) and (**h**) show to the control group that was not exposed to *Reynoutria elliptica* extract. Images (**i**) and (**j**) show *Candida albicans* exposed to 50 μg/mL of *Reynoutria elliptica* extract. Images (**k**) and (**l**) show *Candida albicans* exposed to 200 μg/mL of *Reynoutria elliptica* extract. Magnification factors are indicated at the top of each column.

SEM was used to visualize structural differences for the two types of microbials, relative to those of the control groups (Figure 5). The microbial morphologies of both control groups showed intact and smooth surfaces without cell debris or lysis (Figure 5a,b,g and h). The control group for *S. mutans* exhibited relatively aggregated long chains and differed from the experimental groups in this respect. Additionally, *S. mutans* was formed by the linkage of diplococci, which were composed of a few cocci connected by a wall band. However, for the experimental groups that contained *Reynoutria elliptica* extract, the microbial morphology and density were significantly changed and showed fewer intact cells than did the control group. *Streptococcus mutans* cells treated with *Reynoutria elliptica* extract showed defective morphologies and intracellular contents with rough surfaces and without wall bands. As the concentration of *Reynoutria elliptica* extract was increased, the *S. mutans* cells had small debris and demonstrated loss of their native shape. The control group for *C. albicans* exhibited a larger number of yeast cells than did the 200 μg/mL group. *Candida albicans* cells treated with *Reynoutria elliptica* extract showed visible swelling and obscured surfaces. However, there were no morphological differences for the *C. albicans* cells treated with various concentrations of *Reynoutria elliptica* extract (relative to those in the control group). These results demonstrate that *Reynoutria elliptica* extract significantly inhibited the formation of *S. mutans* and *C. albicans.*

### 3.6. Analysis of Polyphenol and Flavonoid Contents

Increasing the *Reynoutria elliptica* extract concentration significantly increased the content of polyphenol and flavonoid (*p* < 0.05) (Table 1). The contents of polyphenol and flavonoid for the 200 μg/mL group were significantly higher than were those of the other groups (*p* < 0.05). The content of flavonoid for the 100 μg/mL group was not significantly different from that of the 150 μg/mL group (*p* > 0.05).

## 4. Discussion

An ideal antibacterial and antifungal agent for the oral environment must exhibit excellent and selective antibacterial and antifungal action against dental caries, periodontitis, or microorganisms, causing bad breath. In recent years, studies have been carried out on various plant-derived natural extracts that are safe for oral mucosa and can replace antibiotics while continuously acting without side effects [44,45,46]. The *Reynoutria elliptica* extract used in this study, a natural material derived from plants, contains many substances with significant physiological activity, such as phenol compounds, stilbene derivatives, anthraquinone derivatives, and flavonoid compounds [39,47]. Physiological effects of *Reynoutria elliptica* extract, such as anti-inflammatory, antioxidant, anti-hepatitis virus, and antibacterial activities, have been reported. However, studies on antimicrobial efficacy against oral pathogens such as *S. mutans* and *C. albicans* have not been clearly identified.

To the best of our knowledge, our study constitutes the first report of the biocompatibility and antimicrobial effects of *Reynoutria elliptica* extract derived from plants. Therefore, to provide necessary data for the development of oral products using *Reynoutria elliptica* extract to inhibit oral disease, we studied the biocompatibility and antimicrobial properties of *Reynoutria elliptica* extract using various experimental techniques and sought to assess its utility in the dental field.

To probe the effects of various concentrations of *Reynoutria elliptica* extract on cell viability rates, L929 cells were treated with four concentrations of *Reynoutria elliptica* extract, and biocompatibility was evaluated using MTT quantitative analysis according to the method of international standards [48]. As shown in Figure 1, it was observed that the *Reynoutria elliptica* extract produced very weak cytotoxicity at the 200 μg/mL concentration. Additionally, extracts with concentrations lower than 200 μg/mL showed cell viabilities greater than 70%, which meets the criteria for non-cytotoxicity in international standard specifications, and morphological observations also showed no other variations in cells in the experimental groups (as compared to normal L929 cells). Even when treated with *Reynoutria elliptica* extract at concentrations up to 150 μg/mL, cytotoxicity was not observed, and concentrations up to 150 μg/mL are considered to exhibit biocompatibility.

To investigate the effect of *Reynoutria elliptica* extract on the growth of pathogenic microorganisms in the oral cavity, *S. mutans*, bacteria that causes dental caries, and *C. albicans*, a fungus that causes oral candidiasis, were cultured with experimental groups for various time intervals and then absorbance values were determined. As shown in Figure 2, all microbial samples showed the inhibition of microbial growth without significant dependence on the concentration of *Reynoutria elliptica* extract. Conversely, the results of CFU studies showed that the antimicrobial effects seen with various *Reynoutria elliptica* extract concentrations differed for the two microorganisms. Unlike the studies with *S. mutans*, those with *C. albicans* showed significantly higher antifungal activities with higher concentrations of *Reynoutria elliptica* extract. Similar trends were seen in the agar disk diffusion tests conducted in this study. Studies using various concentrations of *Reynoutria elliptica* extract impregnated into paper disks exhibited the formation of transparent circles around the disks, indicating inhibition of the growth of *S. mutans* and *C. albicans*. In addition, the sizes of the transparent circles were found to depend on the concentrations of *Reynoutria elliptica* extract in the disks. As with the CFU studies, higher concentrations in the experimental group with *C. albicans* led to significantly higher antifungal activity. These results suggest that, when using *Reynoutria elliptica* extract for its antimicrobial effects in the oral cavity, the following factors should be considered. If treatment is intended to prevent oral candidiasis, the inhibition of micro fungal activity can be controlled successfully with variations in the concentration of *Reynoutria elliptica* extract. In addition, if it is intended to prevent tooth caries, it may be assumed that any concentration of *Reynoutria elliptica* extract used in this study will show similar antibacterial activity. SEM imaging is a useful method for qualitative evaluation of microbial forms, and we have used it in this study to observe how *Reynoutria elliptica* extract affects the morphology of microorganisms. As can be seen from the SEM results, the *Reynoutria elliptica* extract caused obscured cell bands on *S. mutans*, induced debris on the surface, and interfered with growth, thereby reducing the density of the bacteria. The use of *Reynoutria elliptica* extract with *C. albicans* causes the cells to swell and induces obscured surfaces and interferes with growth, thereby lowering the density of fungi. These results were similar to the results seen for the treatment of *S. mutans* with *Curcuma xanthorrhiza* extract [49], and the effect of *Reynoutria elliptica* extract on the morphological characteristics of pathogenic microorganisms was confirmed in the present study.

The phenolic compounds are secondary metabolites contained in the plant system, and they exhibit physiological activity functions such as antioxidant and antibacterial activities [50,51]. The results of extraction analyses showed that high polyphenol and flavonoid contents were observed as the concentration of the *Reynoutria elliptica* extract was increased. Previous studies have shown an association between phenolic compounds and antimicrobial activity, and it has been reported that there is an inhibitory effect on microorganism growth that is proportional to the content of phenolic compounds contained in plant extracts [52]. Therefore, it is presumed that the polyphenol and flavonoid components contained in the *Reynoutria elliptica* extract used herein have an inhibitory effect on the *S. mutans* and *C. albicans* activities and on their abilities to serve as pathogens in the oral cavity.

When the results of the above studies are combined, our null hypothesis is partially rejected. This study compared the biocompatibility and antimicrobial effects of *Reynoutria elliptica* extract with varying concentrations. In future studies, it will be necessary to expand this study through prolonged long-term comparative studies involving Listerine® ( Johnson & Johnson, New Brunswick, NJ, USA), an oral product based on natural extracts. Listerine^®^ is a mouthwash containing essential oils and active ingredients such as 0.092% eucalyptol, 0.064% thymol, 0.060% methyl salicylate, and 0.042% menthol, and its antibacterial effects have been found in a number of studies [53,54,55]. However, various side effects have been reported, including the poor taste, reversible palatal erythema, burning sensation, and instability of restorative materials resulting from use [56,57,58]. Thus, it is thought that *Reynoutria elliptica* extract, which has low cytotoxicity and can be applied in various concentrations, can be developed as an effective antimicrobial agent against oral pathogens while proving safe for the human body. In this study, since 150 μg/mL concentrations showed no toxicity to L929 cells and showed no significant difference from 200 μg/mL concentrations, it is assumed that the use of oral products using these concentrations would be appropriate for patients suffering from oral diseases such as dental caries or oral candidiasis.

## 5. Conclusions

The following results were obtained by probing cytotoxicity and antimicrobial activity with various *Reynoutria elliptica* extract concentrations.

In cell viability tests, the *Reynoutria elliptica* extract used in this study exhibited a slight cytotoxic effect at a 200 μg/mL concentration, with a viability rate of approximately 60% for L929 cells. However, at concentrations of 150 μg/mL or less, cell viability was 70% or more, this value meets the international standard for biocompatibility. In the antimicrobial tests, antibacterial and antifungal effects exhibited significant differences when compared to the negative control. In particular, *S. mutans* showed an antibacterial effect with no significant difference between experimental groups, regardless of the concentration of the *Reynoutria elliptica* extract. However, *C. albicans* showed an antifungal effect with significant differences depending on the concentration of the *Reynoutria elliptica* extract. In addition, SEM images showed that *Reynoutria elliptica* extract had a significant effect on the morphology and density of the *S. mutans* and *C. albicans* cells.

Overall, the results show that *Reynoutria elliptica* extract at a concentration of 150 μg/mL has excellent antimicrobial effects on *S. mutans* and *C. albicans,* without negatively affecting cell viability and morphology. Accordingly, the results of this research can be used when incorporating *Reynoutria elliptica* extract into oral products that are biologically friendly and capable of controlling oral diseases such as dental caries and oral candidiasis.

## Figures and Tables

**Table 1:** Contents of detected polyphenol and flavonoid in experimental groups. Each value is the average ± standard deviation for 6 measurements.

Experimental Group	Polyphenol Content (μg/mL)	Flavonoid Content (μg/mL)
50 μg/mL	41.2 ± 3.9 ^a^	21.3 ± 1.5 ^a^
100 μg/mL	56.7 ± 2.9 ^b^	24.1 ± 0.9 ^b^
150 μg/mL	65.7 ± 1.7 ^c^	25.4 ± 0.9 ^b^
200 μg/mL	94.0 ± 6.4 ^d^	27.9 ± 2.1 ^c^

The same lowercase letters (**a**–**d**) indicate no differences in polyphenol or flavonoid content among experimental groups within each row (*p* > 0.05).
